# Editorial: Antimicrobial resistant pathogens in sustainable food systems: how to prevent, detect, or control contamination?

**DOI:** 10.3389/fpubh.2023.1221763

**Published:** 2023-06-09

**Authors:** Anibal Concha-Meyer, Magaly Toro, Joseph D. Eifert

**Affiliations:** ^1^Institute of Food Science and Technology (ICYTAL), Austral University of Chile, Valdivia, Chile; ^2^Centro de Estudios en Alimentos Procesados, Talca, Chile; ^3^Joint Institute for Food Safety and Applied Nutrition, University of Maryland, College Park, MD, United States; ^4^Institute of Nutrition and Food Technology, University of Chile, Santiago, Santiago Metropolitan Region (RM), Chile; ^5^Department of Food Science and Technology, Virginia Tech, Blacksburg, VA, United States

**Keywords:** antimicrobial resistance (AMR), surveillance, food pathogens, contamination, food safety

The emergence and spread of antimicrobial resistance (AMR) in bacteria are major public health concerns, posing significant global challenges for sustainable food systems (1). The use of antimicrobial agents in food production, animal-related agriculture, and human medicine is the main driver for the selection of AMR bacteria, which can be transmitted through food or the environment to humans and animals, causing severe infections that may become untreatable with conventional antibiotics (2).

Currently, there is a need for a scientific multidisciplinary and global perspective on the complex challenge of AMR in food systems. Therefore, in March 2022, a special Research Topic, previously proposed to Frontiers, was published titled “Antimicrobial Resistant Pathogens in Sustainable Food Systems: How to Prevent, Detect, or Control Contamination?”. It included a group of immensely committed handling editors and reviewers to supervise the publication of an article collection. This collection of six articles covers a broad range of topics, from the molecular mechanisms of AMR to the development of novel detection methods, risk assessment, and intervention strategies, to prevent and control contamination.

Zhao et al. reviewed the main sources of microbial volatile organic compounds (VOCs) and their potential use as an alternative to synthetic fungicides for post-harvest treatment and control of fruit and vegetable diseases. Furthermore, they discussed recent advancements in understanding the mechanisms of antifungal VOCs and the applications of VOCs produced by antagonistic microorganisms (Zhao et al.).

The study by Eiamsam-Ang et al. presented novel information on the genetic sequencing of *Salmonella* isolates of rare serotypes obtained from farms, slaughterhouses, and retail markets in Thailand between 2011 and 2014. The authors assessed the genetic diversity and relatedness of these isolates. They also characterized their antimicrobial resistance and virulence genes, highlighting the need for appropriate surveillance of food-animal products to reduce public exposure to highly pathogenic, multi-drug resistant *Salmonella* (Eiamsam-Ang et al.).

Research presented by Lambraki et al. investigated the factors influencing AMR in Southeast Asia's food system and proposed intervention measures by integrating local expert perspectives. Their results aim to inform policy improvement and managing decisions (Lambraki et al.).

Mwansa et al. conducted an original cross-sectional study in Zambia, to determine antibiotic resistance patterns of extended-spectrum β-lactamase-producing *E. coli* isolated from stool samples of workers who work at poultry farms and to raise awareness about antibiotic resistance among poultry farmers. The study revealed a high prevalence of AMR linked to tetracycline, trimethoprim/sulfamethoxazole, and ampicillin, among others, in these individuals.

In their contribution, Oguadinma et al. evaluated cross-tolerance to sanitizer among antibiotic-resistant O157:H7 and non-O157:H7 Shiga toxin-producing *E. coli* (STEC). This study highlighted a potential public health concern; antibiotic resistance in *E. coli* could enhance their tolerance to lactic acid and compromise mitigation strategies, such as sanitation, against the pathogen (Oguadinma et al.).

The review article by Kijewska et al. describes the potential of mollusks to become reservoirs of pathogenic bacteria that can carry antibiotic-resistance genes and potentially introduce them into new environments. This finding could expand the existing spectrum of antibiotic-resistance genes present in local biomes (Kijewska et al.).

These contributions provide an overview of current research on AMR in sustainable food systems. They illustrate the multidisciplinary nature of the challenge, requiring collaborative efforts across different sectors and stakeholders, including scientists, policymakers, and industry players.

## Conclusion

In conclusion, this Research Topic highlights the urgent need to address the challenge of AMR in sustainable food systems to safeguard public health, animal welfare, and environmental sustainability. It calls for a concerted and coordinated effort to promote the responsible and prudent use of antimicrobial agents in all sectors, strengthen surveillance and monitoring systems, develop and implement innovative detection and intervention methods, and raise awareness and education on the risks and impacts of AMR.

## Author contributions

AC-M, MT, and JE drafted the editorial, participated in discussions about the ideas, and revised the final editorial. All authors contributed to the article and approved the submitted version.
